# Analysis of cognitive dysfunction and its risk factors in patients with hypertension

**DOI:** 10.1097/MD.0000000000028934

**Published:** 2022-03-11

**Authors:** Xiuping Zhuo, Meinv Huang, Meifang Wu

**Affiliations:** Department of Cardiology, the Affiliated Hospital of Putian University, Putian, China.

**Keywords:** cognitive dysfunction, hypertension, obstructive sleep apnea syndrome

## Abstract

To observe whether obstructive sleep apnea syndrome (OSAS) can aggravate the cognitive dysfunction of patients with hypertension (HTN), and to explore other risk factors.

One hundred one hypertensive patients were selected for information collection. After the polysomnography test, they were divided into HTN-obstructive sleep apnea (OSA) and HTN groups. The Montreal cognitive assessment and the mini-mental state examination scales were used to appraise the patients’ cognitive function. Logistic regressive analysis was used to determine the risk factors of cognitive dysfunction in patients with HTN.

Compared with the HTN patients, HTN-OSA patients performed worse in mini-mental state examination (25.5 ± 2.9 vs 23.5 ± 3.2; *P* = .01) and Montreal cognitive assessment (28 ± 1.58 vs 21.2 ± 3.96; *P* = .003), and patients in the HTN-OSA group seemed more likely to suffer from dementia (31% vs 66%; *P* < .01). The apnea-hypopnea index (AHI) in the HTN group was lower than HTN-OSA group. Through multivariate logistic regression analysis, we can found that alcohol drinking, body mass index, long-term medication, diabetes, hypercholesterolemia, coronary heart disease, and OSAS were the independent risk factors of cognitive dysfunction in patients with HTN.

OSAS can aggravate the cognitive dysfunction of hypertensive patients, besides, drinking, high-body mass index, long-term medication, diabetes, hypercholesterolemia, and coronary heart disease were also the risk factors of cognitive dysfunction in patients with hypertension. The cognitive dysfunction of patients with HTN can benefit from sleep apnea treatment.

## 1 Introduction

Epidemiological studies have shown that: hypertension (HTN) is a parlous factor for a series of adverse consequences, including cognitive decline (the trend of cognitive decline lasts for decades to decades, and only due to age exceeds expectations), cognitive impairment (mild cognitive impairment; memory, thinking, and other cognitive areas have reduced functions, but do not affect daily functions), and dementia (cognitive impairment, including memory and other cognitive areas, but adversely affect daily functions). Compared with normal people, the risk of cognitive dysfunction in HTN patients is increased by about 40%.^[1]^ Therefore, it is urgent to alleviate the cognitive dysfunction of hypertensive patients.

Sleep-related breathing disorders are common, with a prevalence of approximately 10% to 30%. With the development of diagnostic criteria and diagnostic equipment, obstructive sleep apnea syndrome (OSAS) prevalence in the population increases.^[2]^ Studies have found that obstructive sleep apnea (OSA) is particularly common in HTN.^[3]^ OSAS may cause diseases including cardiovascular disease (coronary artery disease, heart failure, atrial fibrillation, HTN, and stroke), metabolic dysfunction, etc.^[4]^ In the sleep cohort in Wisconsin, the increased blood pressure has a linear relationship with the OSAS.^[5]^ In addition to the symptoms of excessive daytime sleepiness and cognitive decline, OSAS is also related to many comorbidities, such as neurodegenerative diseases. Recently, cognitive dysfunction has been found to be one of the consequences of OSAS, so we should pay enough attention.^[6]^ Hence, a high prevalence of OSA and has a great influence on cognitive function in patients with HTN, therefore the purpose of this study was to investigate whether OSA aggravates cognitive impairment in patients with high blood pressure and by the way, to explore other risk factors of cognitive dysfunction in patients with HTN.

## 2 Materials and methods

### 2.1 General information

This was a single-center, retrospective study. Patients with HTN who visited the Department of Cardiology, Putian University, China from February 2019 to February 2020, were included in this study. The inclusion criteria were as follows: determined as a hypertensive patient according to international standards; understand the content of this study, and voluntarily accept sleep monitoring and cognitive function assessment; age ≥18 years old; have good consciousness, thinking, and language communication skills. Exclusion criteria: combined with severe heart, brain, liver, lung, kidney, and other dysfunctions, unable to cooperate with the examination; severe aphasia, confusion, or severe psychiatric complications; central sleep apnea. Finally, a total of 101 patients were enrolled in this study. A smoker was defined as a patient who smoked >1 cigarette per day for >1 year. Drinking in the study was defined as patients who drank alcohol of any type for >14 standard cups (a standard cup is a drink containing 0.6 ounces of alcohol) of alcohol per week or >4 standard cups per day, for at least 1 year. This retrospective observational study was conducted in accordance with the Declaration of Helsinki and approved by the Ethics Committee of the Affiliated Hospital of Putian University. Informed consent was obtained from each participant.

### 2.2 Polysomnography research

All patients underwent polysomnography (PSG) (overnight sleep polysomnography [Alice 5 diagnostic sleep system; Philips Healthcare, Andover, MA]) for 1 night (8 hours) in the sleep laboratory of our department.

### 2.3 Cognitive function assessment

The Montreal cognitive assessment (MoCA) and the mini-mental state examination (MMSE) scale were used to evaluate the cognitive function. If the patient had <12 years of education, the MoCA total score need to add 1 point to correct the deviation.^[7]^ Cognitive assessments were all completed within 10 minutes by the same research team members of the hospital using a face-to-face question-and-answer format. Patients whose MoCA score <26 points and MMSE score <27 points were included in the cognitive impairment group; patients whose MoCA score ≥26 points and MMSE ≥27 points were included in the cognitively normal group. Besides, the incidence of cognitive impairment in patients with OSAS and HTN was calculated.

### 2.4 Statistical analysis methods

Categorical variables were presented as proportion, and comparison was performed by chi-squared test. For normally distributed data, continuous variables were expressed as the mean ± standard deviation and were analyzed using Student *t* test if the variance was homogeneous. If the variance was not uniform, the adjusted *t* test was used. For abnormally distributed data, continuous variables were presented as the median and interquartile range and the Wilcoxon rank-sum test was used. Univariable analysis was performed to assess the risk factors associated with cognitive impairment in HTN patients, and those with a *P*-value of <.20 were incorporated into the multivariable analysis. The results were presented as odds ratios with 95% confidence intervals. *P* < .05 indicates that the difference is significant. The statistical analyses were performed using IBM SPSS Statistics for Windows.

## 3 Results

### 3.1 Study population characteristics

Table 1 presented the baseline characteristics of 101 participants, which summarized demographics, gender distribution, education level, smoking status, underlying diseases, body mass index (BMI), cognitive function, etc. The results showed that men accounted for a significant proportion of the participants, the average age was 52 years old, 63.37% had a drinking habit, 85.14% had a BMI over 23.9, and 62.3% had memory loss.

**Table 1:** Baseline characteristics.

Characteristic	Total (n = 101)
Age in years, median (IQR)	52 (22–86)
Gender (n, %)	
Male	85 (84.2)
Female	16 (15.8)
Cardiovascular risk factors (n, %)	
Diabetes mellitus	11 (10.8)
Hypercholesterolemia	43 (42.6)
Smoking	57 (56.4)
Drink	64 (63.4)
Long-term medication	35 (34.6)
Coronary heart disease	5 (4.9)
Education (yrs, median [IQR])	9 (0–16)
BMI, kg/m^2^ (n, %)	
BMI ≤ 18.5	1 (1)
18.5 ≤ BMI ≤ 23.9	14 (13.9)
BMI ≥ 23.9	86 (85.1)
Type of cognitive disorder (n, %)	
Moderate cognitive impairment	5 (4.8)
Mild cognitive impairment	28 (27.7)
Clinical symptoms (n, %)	
Memory decline	63 (62.3)
Decreased attention	36 (35.6)
Lags in response	35 (34.6)
Other types of symptoms	26 (25.7)

### 3.2 PSG and cognitive function in patients with or without OSA

According to PSG results, they were divided into the HTN group and HTN-OSA group. As shown in Table 2: compared with the HTN group, the SpO2 of the HTN-OSA group was lower (*P* = .001). The total sleep time, median sleep efficiency, and sleep structure of OSA patients and non-OSA patients also were comparable in Table 2. According to the PSG parameters: OSA patients have lower AHI and the lowest SpO2. At the same time, the cognitive function of the 2 groups of patients was tested by the MMSE and MoCA scales. The results showed (Table 3): compared with the HTN group, patients in the HTN-OSA group performed worse in MMSE (25.5 ± 2.9 vs 23.5 ± 3.2; *P* = .01) and MoCA (28 ± 1.58 vs 21.2 ± 3.96; *P* = .003), and based on MMSE and MoCA scores, they are more likely to suffer from dementia (31% vs 66%; *P* < .01).

**Table 2:** Comparision between HTN group and HTN-OSA group.

Characteristic	HTN-OSA (n = 46)	HTN (n = 55)	*P*
Blood oxygen saturation (SpO2, %, median [IQR])	95 (71–98)	97 (95–98)	.001
% TRT SaO2 <90%, median (IQR)	3.7 (0–87.7)	1.2 (0–3.8)	.523
SaO2 <90% time (min, median [IQR])	20.3 (0–576.5)	15.6 (0–430)	.667
Apnea-hypopnea index, median (IQR)	16 (1–73)	0.9 (0–2.7)	.001
Polysomnogram parameters			
Sleep efficiency	77.9 ± 1.7	76.3 ± 1.7	.568
Wake up time after sleep, min	150.6 ± 12.9	160.9 ± 11.9	.59
Total sleep time, min	533.1 ± 12.2	519.2 ± 11.8	.688
Rapid eye movement sleep latency, min	35.2 ± 3.6	44.1 ± 3.8	.487
Non rapid eye movement sleep time, min			
N1 stage	95.4 ± 11.2	95.2 ± 11.1	.369
N2 stage	287.2 ± 10.1	269.2 ± 10	.467
N3 stage	65.9 ± 6	77.2 ± 6.1	.291
SAS severity (n, %)			.137
None (*<*5)	12 (26.1)	36 (65.5)	
Mild (5–15)	20 (43.5)	12 (21.7)	
Moderate (15–30)	5 (10.9)	3 (5.5)	
Severe (*>*30)	9 (19.5)	4 (7.3)	

**Table 3:** The MoCA and MMSE scores.

Group name	HTN	HTN-OSA	*P*
MoCA (‘X ± S)	21.2 ± 3.96	28 ± 1.58	.003
MMSE (‘X ± S)	23.5 ± 3.2	25.5 ± 2.9	.01

### 3.3 Analysis of related factors between patients with cognitive impairment and patients with normal cognition

Then, 101 patients were divided into cognitive normal group and cognitive dysfunction group through the MMSE and MoCA scales and compared the difference between the 2 groups (Table 4). It was found that the occurrence of cognitive dysfunction was related to diabetes, hypercholesterolemia, and hypercholesterolemia, drinking, long-term medication, coronary heart disease, increase in BMI, OSAS (*P* < .05); there was no obvious correlation with gender, age, smoking, and normal cognition group.

**Table 4:** Analysis of related factors between patients with cognitive impairment and patients with normal cognition.

	Cognitive impairment group (n = 33)	Cognitive normal group (n = 68)	χ^2^/*t*	*P*
Male (n, %)	30 (90.9)	55 (80.9)	0.016	.900
Age in years (X ± S)	61.19 ± 7.85	56.89 ± 8.07	0.031	.861
Diabetes mellitus	6 (18.2)	5 (7.4)	8.032	.004
Hypercholesterolemia	21 (63.6)	22 (32.4)	4.321	.03
Smoking	18 (54.6)	39 (57.4)	1.230	.823
Drink	27 (81.8)	37 (54.4)	7.113	.000
Long-term medication	22 (66.7)	13 (19.1)	7.339	.007
Coronary heart disease	3 (9.1)	2 (2.9)	6.466	.000
BMI, kg/m^2^ (n, %)			5.939	.000
BMI ≤ 18.5	1 (3)	0		
18.5 ≤ BMI ≤ 23.9	5 (15.2)	9 (13.2)		
BMI ≥ 23.9	27 (81.8)	59 (86.8)		
OSAS	26 (78.8)	20 (29.4)	8.063	.005

### 3.4 Multivariate logistic regression analysis of cognitive dysfunction

Through multivariate logistic regression analysis (Table 5), it showed that the patient's cognitive impairment was related to alcohol drinking, BMI, long-term medication, and underlying diseases such as diabetes, hypercholesterolemia, beverages, coronary heart disease, and OSAS (*P* < .05).

**Table 5:** Multivariate logistic analysis of cognitive dysfunction.

	*B*	SE	*χ* ^2^	*P*-value	OR 95% CI
Diabetes mellitus	0.119	0.330	1.130	.033	2.888 (0.465–4.652)
Hypercholesterolemia	0.321	0.334	0.921	.037	1.378 (0.716–2.652)
Drink	3.705	0.830	9.931	<.001	4.032 (1.991–7.652)
Long-term medication	1.698	0.541	9.836	.002	5.46 (1.890–15.775)
Coronary heart disease	0.317	0.235	1.820	.017	2.728 (0.459–1.154)
BMI, kg/m^2^	0.140	0.356	3.235	.026	1.958 (1.092–1.837)
OSAS	1.235	0.789	5.326	.001	2.562 (0.563–2.358)

## 4 Discussion

Dementia refers to the gradual decline and irreversibility of cognitive ability that is sufficient to affect related activities of daily living.^[8]^ It influences approximately 50 million people globally, and this population will increase about by 9.9 million every year because of the changes in patient conditions and lack of effective treatments.^[9]^ HTN is the main vascular risk factor for cognitive dysfunction. According to the new guidelines, HTN tortured nearly about 50% of the people in the United States.^[10]^ Because of its central role in cognitive disorders, the World Health Organization set up the HTN 25% reduction in global goal.^[11,12]^ Therefore, we are also working hard to find a solution to this problem.

So far, there are 22 million Americans who undergo sleep apnea, but it is estimated that 80% of men and 93% of women with moderate to severe OSA. Now that the connection between OSA and HTN is noticeable, but the uncertainty about the consequence of this relationship is not consistent.^[13]^ Many studies showed that OSA has nothing to do with HTN, which has raised our doubts about the relationship between OSA and HTN.^[14,15]^ Reports have shown that OSA is significantly relevant to HTN.^[16]^ In addition, OSA is related to the degree of gradual increase in mild OSA, moderate OSA, and severe OSA. This is consistent with the well-recognized relationship between OSA and HTN.^[17,18]^ Our data confirm this association, and our results show that the average MoCA and MMSE scores of HTN-OSAS patients were significantly lower than those of HTN patients. It showed that OSA can exacerbate the cognitive dysfunction of HTN patients. Wang et al^[19]^ pointed out that improving the hypoxic state of OSAS patients is of great significance for improving their cognitive function and delaying the development of cognitive impairment. However, on this basis, implement scientific interventions for patients with controllable risk factors to fundamentally avoid the occurrence of cognitive impairment. This is consistent with our PSG findings: compared with the HTN group, the SpO2 of the HTN+OSAS group was lower than 90%. The total sleep time, median sleep efficiency, and sleep structure of OSA patients and non-OSA patients were comparable to the average SpO2. It is proved that improving OSA can alleviate the cognitive function of hypertensive patients. Then we compared HTN and HTN-OSA patients and found that the occurrence of cognitive dysfunction was significantly related to diabetes, hypercholesterolemia, drinking, long-term medication, coronary heart disease, increase in BMI, OSAS; and gender, age, and smoking were not significantly related to the cognitively normal group. Further through multivariate logistic regression analysis, it showed that the patient's cognitive impairment was connected with alcohol drinking, BMI, long-term medication, and underlying diseases such as diabetes, hypercholesterolemia, beverages, coronary heart disease, and OSAS. It was consistent with the research results of Lal et al,^[20–22]^ indicating that with the increase of patients’ BMI and the existence of underlying diseases or worsening of the disease, the risk of OSAS is higher and blood pressure control is more difficult. The resulting tissue hypoxia and insufficient cerebral perfusion increase the risk of cognitive impairment.

There are several limitations in this study. First, this study investigated HTN and HTN-OSA; however, it does not include only the OSA group. Previous researches reported that the MMSE score of OSA patients is lower than that of healthy controls, however, it is still unclear whether there is a synergistic effect between OSA and HTN. Therefore, in addition to the OSA group, a healthy control group may be needed to affirm this kind of influence in the future. Second, the assessment of cognitive impairment in this study is relatively simple. In the future, it is necessary to conduct an accurate cognitive function assessment to understand the specific impact of OSA on cognitive impairment in HTN patients. Third, the present study was a single-center retrospective study. Hence, the findings of the present study need to be validated through multicenter prospective studies. Besides, the sample size is small which may lead to errors in the results. In summary, our findings cannot be widely applied to the demographic characteristics of HTN patients of different severity. Future studies include more general hypertensive populations that need to be investigated.

## 5 Conclusion

Our research showed that OSA significantly causes cognitive dysfunction in HTN patients. In addition, the occurrence of cognitive dysfunction in HTN patients is significantly related to diabetes, hypercholesterolemia, drinking, long-term medication, coronary heart disease, and increased BMI. It is worth noting that in the future, in HTN patients with OSA, in addition to treating the corresponding underlying diseases, appropriate anti-OSA treatment may improve cognitive function, promote functional recovery, and increase the quality of life of HTN patients.

## Author contributions

Xiuping Zhuo, Meinv Huang, and Meifang Wu designed the study, collected the data, analyzed the relevant information, wrote the manuscript and approved the final submission.

**Conceptualization:** Xiuping Zhuo.

**Formal analysis:** Xiuping Zhuo, Meifang Wu.

**Investigation:** Meifang Wu.

**Methodology:** Xiuping Zhuo, Meifang Wu.

**Project administration:** Xiuping Zhuo, Meinv Huang, Meifang Wu.

**Resources:** Meinv Huang.

**Software:** Meinv Huang, Meifang Wu.

**Supervision:** Meinv Huang, Meifang Wu.

**Visualization:** Xiuping Zhuo, Meinv Huang, Meifang Wu.

**Writing – original draft:** Xiuping Zhuo, Meinv Huang, Meifang Wu.

**Writing – review & editing:** Xiuping Zhuo, Meinv Huang, Meifang Wu.

## Correction

When originally published, the Affiliated Hospital of Putian University appeared incorrectly throughout the article as the Putian University. It has been corrected.
